# Qigong for Muscle Strength and Static Postural Control in Middle-Aged and Older Postmenopausal Women: A Randomized Controlled Trial

**DOI:** 10.3389/fmed.2021.784320

**Published:** 2021-12-08

**Authors:** María del Carmen Carcelén-Fraile, Agustín Aibar-Almazán, Antonio Martínez-Amat, Vânia Brandão-Loureiro, José Daniel Jiménez-García, Yolanda Castellote-Caballero, Fidel Hita-Contreras

**Affiliations:** ^1^Department of Health Sciences, Faculty of Health Sciences, University of Jaén, Jaén, Spain; ^2^Escola Superior de Educação, Instituto Politécnico de Beja, Beja, Portugal; ^3^Department of Physical Education, Faculty of Education Sciences, University of Cádiz, Cádiz, Spain

**Keywords:** postmenopausal, middle-aged, Qigong, muscle strength, postural control

## Abstract

In the present study, we aimed to determine the effects of a Qigong exercise program on the muscle strength and postural control in middle-aged and older postmenopausal women. This is a randomized clinical trial (https://clinicaltrials.gov/ct2/show/NCT03989453) conducted on 125 women who were initially assigned to either an experimental group (*n* = 63) that performed a Qigong exercise program for 12 weeks or to a control group (*n* = 62) that did not receive any intervention. Muscle strength (dynamometer) and postural control (stabilometric platform) were evaluated before and immediately after an intervention period. The main findings of this study suggest that the women in the experimental group had improvements in muscle strength, mean velocity of the displacement of the center of pressure (CoP) with both eyes open and closed, and the surface sway area covered by the CoP, as well as the mediolateral and anteroposterior oscillations of the CoP, only with eyes open. The results of the present study determined that a 12 week Qigong exercise program has beneficial effects on muscle strength and postural control of middle-aged and older postmenopausal Spanish women.

## Introduction

With aging, bone mineral density, muscle mass and strength gradually decrease and the menopause onset accelerates this process (1). Sarcopenia has long been associated with older people, but its development is recognized to begin earlier in life, and, in order to prevent or delay its appearance, strategies should also focus on middle age people (2, 3). In 2018, the European Working Group on Sarcopenia In older people (EWGSOP2) used low muscle strength as the primary parameter to define sarcopenia (3). Low muscle strength can lead to a loss of functional capacity, becoming one of the main causes of a disability (4). Similarly, women with decreased muscle mass become susceptible to a series of problems that affects postural function, which causes a series of changes in gait and reduced postural control through increased pressure velocity and center of oscillation (5). The result of this postural instability and loss of muscle strength can lead to a decrease in physical function (6). All these factors can lead to an increase in the risk of fall and fall-related injuries, which is a major health problem for older people, especially women, due to the menopause-related osteoporosis.

Physical activity has been shown to be beneficial for the physical and psychological health of postmenopausal women (7) and, in recent decades, has become one of the non-pharmacological interventions most used by women (8). Qigong is considered to be one of the most popular health preservation and disease prevention strategies, based mainly on the work of energy or the movement of energy (9). The term Qigong is a neologism extended in the 1950's that encompasses a series of techniques with the aim of improving physical, mental, emotional, and respiratory well-being, and is one of the four pillars of traditional Chinese medicine (10). This method of exercise is based on a series of natural principles that gives meaning to its purpose: concentration, considered to be the active mental component of an activity (11); relaxation which refers to the neuromuscular and endocrine condition of the body and also to the emotional and mental form (12); breathing which is closely related to Qi (13); body posture, which plays a very important role in the functioning of all aspects of the physiological process (14); and movement, since slow and repetitive movements facilitate the function of the autonomic nervous system by reducing the activity of the sympathetic nervous system and increasing the activity of the parasympathetic nervous system (11). Unlike conventional exercise, Qigong aims to improve physical condition and general well-being through coordinated rhythmic movements, regulated breathing, and meditation (15). The practice of Qigong exercise can help to maintain satisfactory health and to prevent as well as treat disease (16), however, the physiological effects of Qigong exercise in healthy older women have not been reported.

Taking into consideration the above, the objective of this study was to study the effects of a Qigong exercise program on the muscle strength and postural control of middle-aged and older postmenopausal Spanish women. We hypothesized that women who carried out a twelve week Qigong program would increase handgrip strength and improve posturographic parameters.

## Materials and Methods

### Study Design

The present randomized and controlled clinical trial aimed to analyze the effects of a 12 week Qigong exercise program on the muscle strength and postural control of middle-aged and older postmenopausal Spanish women (https://clinicaltrials.gov/ct2/show/NCT03989453). All the women who participated in the study signed an informed consent approved by the Human Ethics Committee of the University of Jaén. The study was conducted in accordance with the Declaration of Helsinki, good clinical practice, and applicable laws and regulations.

### Participants

**Figure 1:**
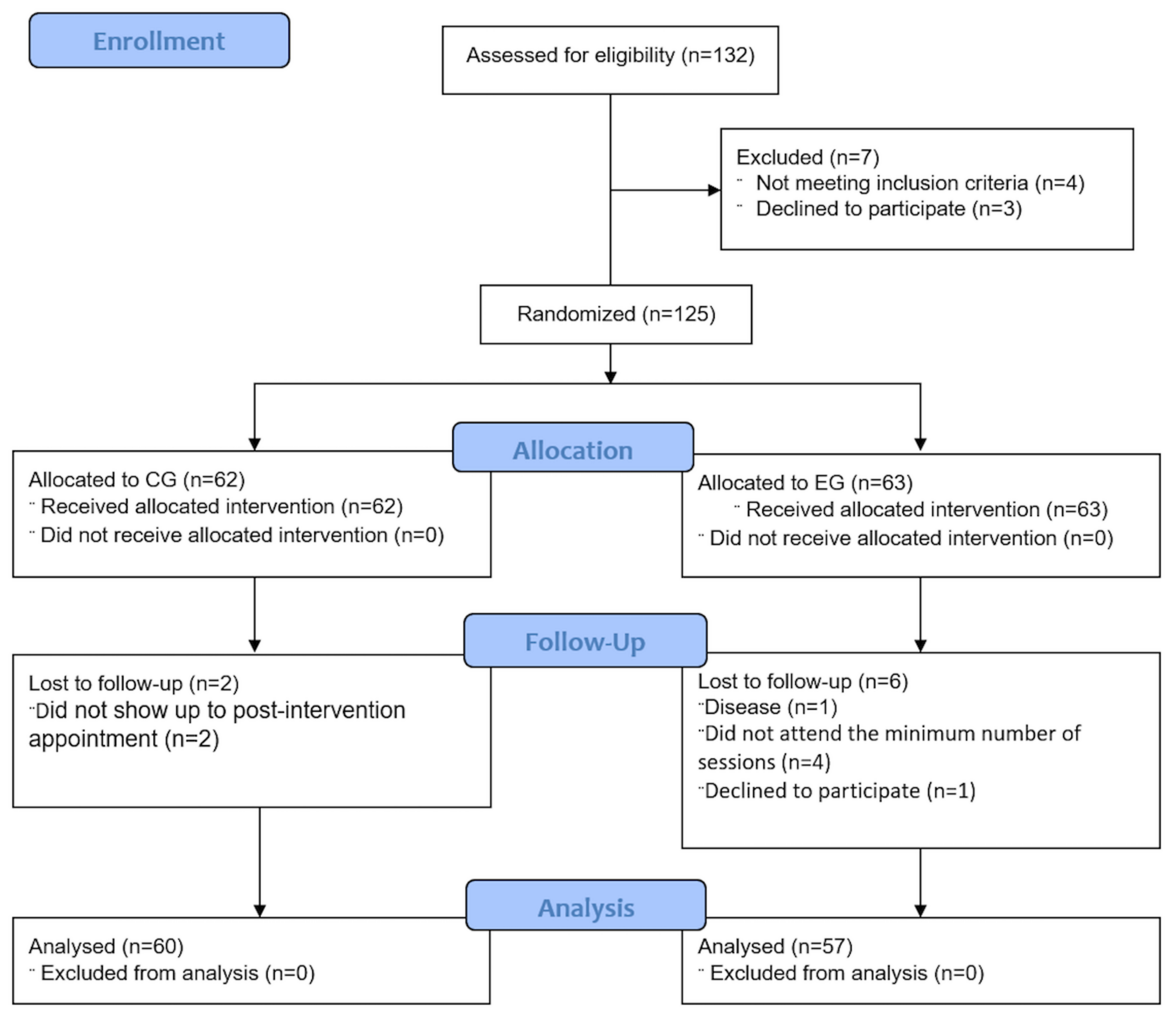
Flow diagram of study design.

All the participants were recruited from two active participation centers in the city of Jaén (Spain). Initially, 132 women were contacted, of which 125 women met the eligibility criteria and accepted their participation in the study (Figure 1). Women who were included (i) had amenorrhea for at least 12 months; (ii) were able to understand and complete each of the self-administered questionnaires, as well as to carry out the tests required to obtain the variables of this study; and (iii) could understand and were able to carry out the instructions, activities, and protocols related to the Qigong exercise program. Participants who were excluded were (i) under menopausal hormone therapy; (ii) suffered from some type of vestibular disease or disorder; (iii) had some type of systemic disease (for example, musculoskeletal, neurodegenerative, or vision type) that made it impossible for them to perform the postural balance test or Qigong exercises; or (iv) were taking drugs that impaired the central nervous system, coordination, or balance (e.g., anxiolytics, antidepressants, or vestibular sedatives).

### Randomization

The women who participated in the study were assigned to an experimental group (EG) or a control group (CG) at a 1:1 ratio, using a table of random numbers produced by a computer. For this, closed opaque envelopes were used, and the assignment of groups was carried out by an independent party not related to the selection of participants, the intervention, or data collection. Finally, 63 women were assigned to the EG and 62 women were assigned to the CG.

### Intervention

The women included in the experimental group participated in a Baduanjin Qigong exercise program which was comprised of two sessions a week for 12 weeks, for a total of 24 sessions. Each session included a 10 min warm-up portion, a 40 min main portion, and a 15 min cool-down portion, for a total of 60 min. For the warm-up process breathing, stretching, and joint mobility exercises were performed; the cooling process was composed of flexibility and stretching exercises. The main portion consisted of 8 Baduanjin Qigong postures known as “the eight pieces of brocade”: (1) “Shuang Shou Tuo Tian Li San Jiao” (holding the sky), (2) “Zuo You Kai Gong Si She Diao” (make a bow on both sides like shooting an eagle), (3) “Tiao Li Pi Wei Xu Dan Ju” (separate heaven and earth), (4) “Wu Lao Qi Shang Wang Hou Qiao” (look back), (5) “Yao Tou Bai Wei Qu Xin Huo” (move the head and shake the tail), (6) “Liang Shou Pan Zu Gu Shen Yao” (touch the feet with both hands), (7) “Cuan Quan Nu Mu Zeng Qi Li” (clenching fists with angry eyes), (8) “Bei Hou Qi Dian Bai Bing Xiao” (raising and lowering the heels). The 8 postures were preceded by a preparation posture and finished with a final posture, for a total of 10 postures. In contrast, the women assigned to the control group maintained their daily activities and did not participate in any exercise program. In addition, they were provided with a series of recommendations in order to promote physical activity (http://www.juntadeandalucia.es/salud/servicios/contenidos/andaluciaessalud/docs/130/GuiaRecomendaciones_AF.pdf).

### Outcomes

All data and variables in the present study were obtained just before group assignment and after the intervention period. Sociodemographic and clinical data were collected such as age, weight (Tefal precision digital weight scale 100 g−130 kg), height (Asimed T201-T4 height rod), and history of falls in the last year. For the evaluation of body mass index (BMI), each participant's weight (kg) was divided by their height squared (m2) (17).

#### Muscle Strength

To evaluate muscle strength, handgrip strength was calculated using a TKK 5001, Grip-A, handheld analog dynamometer (Takei, Tokyo, Japan). Handgrip strength is a widely used variable among older people that has been shown to correlate with various health indicators and lower limb strength (18, 19). To obtain it, the participants were asked to apply their maximum handgrip strength on three occasions with both hands (total of six), with rest periods of 30 s between each measurement, using the maximum value obtained for this study.

#### Static Postural Control

For the study of static postural control, a stabilometric platform (Sensor Medica, Rome, Italy) and the FreeStep © Standard 3.0 software (Sensor Medica, Rome, Italy) were used, which has been previously used in this type of population (20). This analysis focuses on the displacements of the center of pressure (CoP) and provides a series of variables or posturographic parameters. The CoP displacement is regarded as the most reliable parameter to evaluate postural balance control under static conditions. In this study, the following were analyzed: the mean velocity (V) of the CoP displacements (m/s), the sway area (S) covered by the CoP (cm2), and the mean value of the mediolateral (X) and anteroposterior (Y) displacements of the CoP (mm). To obtain these parameters, the Romberg (21) test was used with test duration of 30 s. The participants stood barefoot on the stabilometric platform, with their heels 2 cm apart and their feet spread out at a 30° forward angle, their arms relaxed resting on either side of the body, and their gaze fixed on a point located about 2.5 m ahead. The Romberg test was performed twice consecutively, once with eyes open (EO) and a second time with eyes closed (EC), leaving 1 min of rest between both tests.

### Sample Size Calculation

The sample size was calculated through Jan 3 (GlaxoSmithKline, SA, Madrid, Spain). The required sample was determined using Liao et al. (22) as a reference. To obtain a statistically significant difference using the muscle strength score obtained as a dependent variable, with a power of 0.80, a significance level of 95%, and considering an estimated dropout of 10%, a minimum of 35 experimental units were required in the group reference and 43 units in the experimental group, totaling 78 experimental units in the study.

### Statistical Analysis

The statistical analysis was carried out using the SPSS statistical program, version 20.0 for Windows (SPSS, Inc., Chicago, IL, USA). We worked with a level of statistical significance of *p*-value <0.05. The continuous variables were presented by means and standard deviations and the categorical variables in frequencies and percentages. The Kolmogorov–Smirnov test was used to check the normality of the data distribution. To determine the possible differences between both study groups before the start of the study, a Student's *t*-test and chi-square test were used for continuous and categorical variables, respectively. To analyze the possible differences in values between the variables studied, a mixed analysis of variance was carried out, in which the study group was considered to be the intergroup factor (CG vs. EG), and the measurement time of the variables (pre- and post-intervention) the intragroup factor. The dependent variables were static postural control through posturography and muscle strength through dynamometer. All the analyses were carried out independently for each dependent variable and the possible interactions “group x measurement time” were analyzed. To assess the effect size of the possible intergroup and intragroup differences, the Cohen's d statistic was used. Values of <0.2 indicated an insignificant effect size, between ≥0.2 and <0.5 a small effect size, between ≥0.5 and <0.8 a medium effect size, and values ≥0.8 a large effect size (23).

## Results

Table 1 shows the descriptive information of the women at the beginning of the study, in which it can be observed that no significant differences were found between the groups. All women participated in at least 90.8% of the sessions and no injuries or adverse effects were reported during the course of the intervention.

**Table 1:** Baseline characteristics of study participants.

		**Total**	**EG**	**CG**	***P*-value**
		**(*n* = 117)**	**(*n* = 57)**	**(*n* =60)**	
Age (years)		69.73 ± 6.44	69.70 ± 6.15	69.75 ± 6.76	0.968
Height (m)		156 ± 0.05	157 ± 0.06	155 ± 0.05	0.205
Weight (Kg)		65.42 ± 7.82	66.72 ± 7.85	64.19 ± 7.65	0.800
BMI (kg/m^2^)		26.83 ± 2.53	27.15 ± 2.67	26.53 ± 2.38	0.187
History of falls	No	81 (69.23)	42 (51.90)	39 (48.10)	0.313
	Yes	36 (30.67)	15 (41.70)	21 (58.30)	
Muscle strength		16.61 ± 3.87	16.25 ± 3.82	16.95 ± 3.92	0.336
VEO		16.34 ± 3.83	15.76 ± 3.87	16.89 ± 3.81	0.115
SEO		146.69 ± 152.04	132.87 ± 137.55	159.82 ± 164.71	0.340
XEO		0.58 ± 0.20	0.58 ± 0.22	0.57 ± 0.19	0.739
YEO		0.41 ± 0.16	0.40 ± 0.17	0.41 ± 0.15	0.914
VEC		15.22 ± 4.18	14.89 ± 4.35	15.54 ± 4.02	0.406
SEC		102.59 ± 103.95	105.18 ± 117.81	100.13 ± 89.77	0.794
XEC		0.58 ± 0.16	0.56 ± 0.16	0.60 ± 0.16	0.128
YEC		0.43 ± 0.16	0.42 ± 0.12	0.43 ± 0.20	0.599

*Data are expressed as mean and standard deviation (continuous variables) and frequency and percentage (categorical variables). EG, experimental group; CG, control group; BMI, Body Mass Index; V, velocity of the center of pressure displacements (mm/s^2^); V, sway area of the center of pressure position; X, mediolateral displacements of the center of pressure; Y = anteroposterior displacements of the center of pressure; EO, eyes open; EC, eyes closed*.

### Muscle Strength

Table 2 shows the main effects on muscle strength. According to our results, higher (and therefore better) scores were obtained on the dynamometer test after the Qigong exercise program (t _(56)_ = −11.525, *p* < 0.001, Cohen's d = 0.30). In addition, differences were observed between the groups after the intervention (t _(115)_ = −2.295, *p* = 0.024, Co-hen's d = 0.42).

**Table 2:** Effects of Qigong training on muscle stregth and postural control.

	**Pre-intervention**		**Post-intervention**		**Group**			**Time**			**Group x Time**		
	**EG**	**CG**	**EG**	**CG**	**F (1.115)**	***P*-value**	**η ^**2**^**	**F (1.115)**	***P*-value**	**η ^**2**^**	**F (1.115)**	***P*-value**	**η ^**2**^**
Muscle strength	16.25 ± 3.82	16.95 ± 3.92	17.39 ± 3.71	15.91 ± 3.29	0.344	0.559	0.003	0.327	0.568	0.003	141.825	<0.001	0.552
VEO	15.76 ± 3.87	16.89 ± 3.81	14.63 ± 4.02	18.21 ± 5.07	14.297	<0.001	0.111	0.035	0.851	0.000	87.934	0.011	0.055
SEO	132.87 ± 137.55	159.82 ± 164.71	115.78 ± 105.98	162.80 ± 132.08	2.210	0.140	0.019	2.128	0.147	0.018	4.304	0.040	0.036
XEO	0.58 ± 0.22	0.57 ± 0.19	0.40 ± 0.17	0.51 ± 0.21	2.580	0.111	0.022	38.133	<0.001	0.249	9.135	0.003	0.074
YEO	0.40 ± 0.17	0.41 ± 0.15	0.36 ± 0.16	0.46 ± 0.17	5.206	0.024	0.043	0.019	0.891	0.000	7.893	0.006	0.064
VEC	14.89 ± 4.35	15.54 ± 4.02	13.23 ± 5.52	16.75 ± 7.02	6.802	0.010	0.056	0.144	0.705	0.001	5.982	0.016	0.049
SEC	105.18 ± 117.81	100.13 ± 89.77	91.73 ± 75.63	82.96 ± 68.84	0.198	0.658	0.002	6.895	0.010	0.057	0.102	0.750	0.001
XEC	0.56 ± 0.16	0.60 ± 0.16	0.51 ± 0.20	0.62 ± 0.22	8.287	0.005	0.067	0.401	0.528	0.003	2.537	0.114	0.022
YEC	0.42 ± 0.12	0.43 ± 0.20	0.36 ± 0.13	0.43 ± 0.17	3.646	0.059	0.031	2.242	0.137	0.019	2.270	0.135	0.019

*The quantitative variables are presented as mean and standard deviation. Qualitative variables are presented as frequency and percentage. EG, experimental group; CG, control group; V, velocity of the center of pressure displacements (mm/s^2^); S, sway area covered by the center of pressure; X, mediolateral displacements of the center of pressure; Y, anteroposterior displacements of the center of pressure; EO, eyes open; EC, eyes closed*.

### Static Postural Control

Regarding static postural control (Table 2), a significant main effect for the group variable, and for the group x time interaction were observed in both VEO and VEC. The detailed analysis of the interaction showed significant differences within the EG both with eyes open (t _(56)_ = 2.011, *p* = 0.049, Cohen's d = 0.29) and with eyes closed (t _(56)_ = 2.012, *p* = 0.049, Cohen's d = 0.33). In addition, differences between the groups could be observed after the intervention for both eyes open and eyes closed conditions (t _(115)_ = 4.218, *p* < 0.001 Cohen's d = 0.78 and t _(115)_ = 3.009, *p* = 0.003, Cohen's d = 0.56, respectively).

As for the sway area covered by the CoP, there was a significant main effect for the time variable only in SEC, while a significant group x time interaction was observed only in SEO. The specific analysis of this group x time interaction showed significant differences between the pre and post measurements in the experimental group (t _(56)_ = 2.314, *p* = 0.024, Cohen's d = 0.14) and between both groups in the post-intervention measurement (t _(115)_ = 2.117, *p* = 0.036, Cohen's d = 0.39).

Regarding the mediolateral oscillations of the CoP, results revealed a main effect in the group variable only for XEC, and in the time variable only for XEO, which was the only one that showed a significant group x time interaction. The analysis of this interaction revealed significant between groups differences with XEO in the post-intervention measure (t _(115)_ = 3.118, *p* = 0.002, Cohen's d = 0.58) and significant within-group differences for XEO in women who performed Qigong exercises (t _(56)_ = 8.742, *p* < 0.001, Co-hen's d = 0.94).

Finally, in the analysis of the anteroposterior oscillations of the CoP, only YOA exhibited significant results (main effect in the group variable and group x time interaction). The analysis of this interaction revealed that statistically significant within-group differences in the EG (t _(56)_ = 2.099, *p* = 0.040, Cohen's d = 0.24) and between both groups in the post-intervention measurement (t _(115)_ = 3.445, *p* = 0.001, Cohen's d = 0.61). On the contrary, there were no significant results regarding YEC.

## Discussion

In the present study, we aimed to analyze the effects of a 12 week Qigong-based exercise program on the static postural control and muscle strength of middle-aged and older Spanish women. Our findings showed that static postural control and muscle strength improved significantly after the Qigong intervention.

Regardless of weight variations, menopause has been shown to be associated with significant changes in body composition that can affect physical function, including muscle strength, which is essential for the successful performance of activities of daily living (24). Epidemiological studies have shown that low handgrip strength in healthy adults is a risk factor for functional limitations and disability in old age, as well as mortality from all causes (25, 26). Physical exercise is one of the main non-pharmacological treatments for the maintenance or improvement of muscle strength (27). In addition, physical exercise improves mobility, functional capacity, and therefore personal autonomy (28).

Our findings showed that the handgrip strength of the 12 week Baduanjin-based training group was significantly higher after the intervention, and also as compared with the control group at the post-intervention assessment. Our result was in accordance to that of Liu et al. (29), who verified the positive effects of Baduanjin on handgrip strength, but, unlike our work, their study involved frail older adults and did not use comparisons with a control group, and to the study by Peng et al. (30) in which they observed improvements in grip strength in the group that carried out a Baduanjin training for 12 weeks but in a sample of university students. In contrast, Tsai et al. (31) found no statistically significant differences in grip strength of middle-aged women, after 8 weeks of another Chi Kung modality known as Ching Ching. Along the same lines, several studies have observed benefits in handgrip strength, but carried out different types of physical training for postmenopausal women. One of these studies conducted a randomized controlled clinical trial and found improvements in handgrip strength after a 12 week Pilates exercise program (32). Another study (33) found improvements in grip strength after performing a resistance exercise program for 12 weeks. Finally, in a study by Moreira et al. (34) statistically significant differences in handgrip strength were observed in the group that participated in training based on high intensity aquatic exercises for 24 weeks. The present study provides new data on the effects of other types of training such as Baduanjin on the handgrip strength of postmenopausal women, a growing population.

Stability and static postural balance decrease with age. Loss of balance and increased body sway are important risk factors for falls in postmenopausal women. An evaluation of postural balance is essential to develop effective preventive actions regarding falls, as well as improvements in the quality of life of these women (35). The static condition refers to balance under unperturbed environments such as quiet standing (36), and in the present study, a stabilometric platform was used to objectively assess static postural control, which is considered to be the gold standard for assessing postural balance (37). The benefits of programmed exercise on static postural control have been described in the elderly; Wang et al. (38), in a cross-sectional study conducted in older adults, concluded that long-term Tai Chi and jogging contributed to static balance control, and Forte et al. (39) have shown that gross-motor skill exercise and strength training may improve both static postural balance and dynamic functional balance in healthy older adults. However, Chen at al. (40) did not find significant changes in the CoP measurements in older people after 12 weeks of Tai Chi. The central nervous system and the musculoskeletal system are the main physiological and neurological processes involved in postural control (41). Qigong exercises are a strategy that encompasses all the fundamental aspects of the balance system, since these exercises train the sensory systems related to postural stability and balance (42). Moreover, it has been suggested that a Qigong exercise-based training program improved the use of visual and vestibular information, two mechanisms by which training can improve balance (43). Our analysis showed that participants who carried out a Baduanjin program experienced statistically significant improvements, under both EO and EC conditions, regarding the mean velocity of the CoP, which is considered the most accurate measure for the evaluation of postural balance (44) and an independent predictor of the incidence of falls and fractures (45). Improvements in XEO, YEO and SEO were observed after the 12 week training. Taken together, the positive effects of Baduanjin training on static postural control can be also explained by the characteristic of dynamic movements. When performing the exercises, the participants were asked to control their balance (center of gravity) while performing gentle movements with the lumbar spine as the axis. Specifically, weight shift in four directions, especially of the upper body, constantly challenged the postural stability of the lower extremities. For example, when performing exercise five, individuals were required to circle the lumbar spine at approximately 45 degrees, while exercise six required a 90-degree forward tilt of the upper body. By performing these unique movements, the participants were trained to maintain their center of gravity using their lower limbs, especially their feet that must be rooted on the ground, without crossing the shoulder width limit.

Although there have been previous studies that have linked this exercise program with postural control, there are no studies that have proven such benefits in healthy postmenopausal women. For example, Liu et al. (46) found that static balance, measured by a one-legged standing test, improved in a sample group of Chinese older adults living in a country community that carried out an intervention for 12 weeks, but due to the de-sign of their study (quasi-experimental), they did not make comparisons with a control group. Likewise, the effects of other types of training to improve postural control have been verified. For example, in a study by Choi et al. (47) who carried out a Tai Chi exercise program for 12 weeks with a population of people older than 60 years, their results showed significant improvements with their eyes open, but not with eyes closed.

This study has a number of limitations. Only short-term effects were assessed and this study was carried out with women living in the community, and therefore our conclusions cannot be extended to the general older population. In addition, although the participants were blinded to the hypotheses, they were not blinded to the intervention due to the nature of the study. Future studies should be conducted that consider the medium- and long-term effects on both older men and women.

## Conclusions

In conclusion, the present study shows that a 12 week Qigong exercise program had beneficial effects on the muscle strength of middle-aged and older postmenopausal Spanish women. Improvements were also observed in static postural control, specifically, in the mean velocity of the CoP displacements, with both eyes open and closed, as well as in the sway area covered by the CoP, the mediolateral and the anteroposterior oscillations of the CoP, all under eyes open condition.

## Data Availability Statement

The original contributions presented in the study are included in the article/supplementary material, additional queries can be directed to the corresponding authors.

## Ethics Statement

The studies involving human participants were reviewed and approved by University of Jaén. The patients/participants provided their written informed consent to participate in this study.

## Author Contributions

MC-F and FH-C: conceptualization. AA-A, VB-L, and YC-C: methodology. AA-A and JJ-G: formal analysis. MC-F and JJ-G: writing—original draft preparation. VB-L, MC-F, and YC-C: writing—review and editing. AA-A, FH-C, and AM-A: supervision. FH-C and AM-A: funding acquisition. All authors have read and agreed to the published version of the manuscript.

## Funding

This work was partly supported by project 1260735 from the 2014–2020 Operational Programme FEDER in Andalusia and by the project UP Again Senior (PNDPT - IPDJ - CP/532/DDT/2020) from the Instituto Português do Desporto e Juventude.

## Conflict of Interest

The authors declare that the research was conducted in the absence of any commercial or financial relationships that could be construed as a potential conflict of interest.

## Publisher's Note

All claims expressed in this article are solely those of the authors and do not necessarily represent those of their affiliated organizations, or those of the publisher, the editors and the reviewers. Any product that may be evaluated in this article, or claim that may be made by its manufacturer, is not guaranteed or endorsed by the publisher.
